# Use of Interrupted Time-Series Method to Evaluate the Impact of Cigarette Excise Tax Increases in Pennsylvania, 2000–2009

**DOI:** 10.5888/pcd10.120268

**Published:** 2013-10-03

**Authors:** Zhen-qiang Ma, Lewis H. Kuller, Monica A. Fisher, Stephen M. Ostroff

**Affiliations:** Author Affiliations: Lewis H. Kuller, Department of Epidemiology, University of Pittsburgh, Pittsburgh, Pennsylvania; Monica A. Fisher, Stephen M. Ostroff, Pennsylvania Department of Health, Bureau of Epidemiology, Harrisburg, Pennsylvania.

## Abstract

**Introduction:**

Scientific evidence shows that cigarette price increases can significantly reduce smoking prevalence and smoking initiation among adolescents and young adults. However, data are lacking regarding the effectiveness of increasing Pennsylvania’s cigarette tax to reduce smoking and/or adverse health effects of smoking. The objective of our study was to assess the impact of cigarette tax increases and resulting price increases on smoking prevalence, acute myocardial infarction (AMI) and asthma hospitalization rates, and sudden cardiac death (SCD) rates in Pennsylvania.

**Methods:**

We used segmented regression analyses of interrupted time series to evaluate the level and trend changes in Pennsylvania adults’ current smoking prevalence, age-adjusted AMI and asthma hospitalization rates, age-specific asthma hospitalization rates, and age-adjusted SCD rates following 2 cigarette excise tax increases.

**Results:**

After the first excise tax increase, no beneficial effects were noted on the outcomes of interest. The second tax increase was associated with significant declines in smoking prevalence for people aged 18 to 39, age-adjusted AMI hospitalization rates for men, age-adjusted asthma hospitalizations rates, and SCD rates among men. Overall smoking prevalence declined by 5.2% (*P* = .01), with a quarterly decrease of 1.4% (*P* = .01) for people aged 18 to 39 years. The age-adjusted AMI hospitalization rate for men showed a decline of 3.87/100,000 population (*P* = .04). The rate of age-adjusted asthma hospitalizations decreased by 10.05/100,000 population (*P* < .001), and the quarterly trend decreased by 3.21/100,000 population (*P* < .001). Quarterly SCD rates for men decreased by 1.34/100,000 population (*P* < .001).

**Conclusion:**

An increase in the price of cigarettes to more than $4 per 20-cigarette pack was associated with a significant decrease in smoking among younger people (aged 18–39). Decreases were also seen in asthma hospitalizations and men’s age-adjusted AMI hospitalization and SCD rates. Further research and policy development regarding the effect of cigarette taxes on tobacco consumption should be cognizant of the psychological tipping points at which overall price affects smoking patterns.

## Introduction

Cigarette smoking is well-documented as the most important cause of chronic bronchitis in the United States and as a risk factor for coronary heart disease (1,2). A 2009 report by the Institute of Medicine (IOM) concluded that secondhand smoke exposure increases the risk of coronary heart disease by 25% to 30%, and smoking bans that reduce exposure to indoor secondhand smoke are associated with a decreased incidence of acute myocardial infarction (AMI) (3). Actively smoking or being exposed to secondhand smoke can trigger an asthma attack and worsen asthma symptoms. Despite numerous health studies and scientific reports identifying the harmful effects of smoking and the benefit of smoking cessation, 18.4% of Pennsylvania adults smoked in 2010 (4), a prevalence substantially higher than the national *Healthy People 2020* objective of 12.0% (5).

Numerous economic studies have documented that cigarette price increases, most often through cigarette excise taxes, are significantly associated with reductions in smoking prevalence and smoking initiation (2,6,7). One study documented that a 10% increase in cigarette price was associated with reductions of an estimated 3% to 5% in overall short-term cigarette consumption for adults (6). Cigarette tax increases can also achieve long-term smoking reductions because they disproportionately affect the smoking prevalence among younger adults (7).

On July 15, 2002, Pennsylvania raised its cigarette excise tax by $0.69, from $0.31 to $1.00 per pack. On January 7, 2004, the cigarette excise tax was raised again, this time by $0.35, from $1.00 to $1.35 pack. Although states generally increase cigarette excise taxes to raise revenues and provide funding for state tobacco-control programs (8), indirect effects of these measures include a reduction in smoking consumption (prevalence and number of cigarettes smoked) and tobacco-related health consequences. 

The objective of our study was to assess the impact in Pennsylvania of 2 increases in cigarette excise tax and the resulting price increase on either an immediate reduction or long-term change in the trend of smoking prevalence, AMI and asthma hospitalization rates, and sudden cardiac death (SCD) rates. These health conditions were chosen because they have been clearly linked to exposure to tobacco smoke.

## Methods

### Data sources

We evaluated the overall and age-specific quarterly Pennsylvania adult smoking prevalence by using 1998–2010 Behavioral Risk Factor Surveillance System (BRFSS) survey data (4). We used the 2000–2009 Pennsylvania Health Care Cost Containment Council (PHC4) hospital discharge database (9) to evaluate AMI and asthma quarterly hospitalization rates. Pennsylvania 2000–2009 vital statistics death-certificate data (10) were used to evaluate SCD quarterly age-adjusted rates. Cigarette-related tax revenue and tax history information were obtained from the Pennsylvania Department of Revenue (11). Pennsylvania population data were derived from the Population Division of the US Census Bureau (12).

### Statistical analysis

Quarterly Pennsylvania adult current smoking prevalence was calculated from the 1998–2010 Pennsylvania BRFSS (4). The Pennsylvania BRFSS is a multistage stratified population sample survey. Survey weight and stratification were used to calculate the smoking prevalence; the interview month was used to determine the quarter of the year. Pennsylvania BRFSS smoking prevalence was examined for adults aged 18 to 39 and 40 or older.

Quarterly age-adjusted AMI and asthma hospitalization rates were calculated from 2000 through 2009 PHC4 hospital discharge data, using the US 2000 standard population. AMI was defined as a primary discharge diagnosis (International Classification of Diseases, Ninth Revision, Clinical Modification [ICD-9-CM] code 410). Other subacute and chronic ischemic heart diseases (ICD-9-CM codes 411–414) were not included in this analysis. Asthma was defined by the primary discharge diagnosis (ICD-9-CM code 493). Both AMI and asthma hospitalization rates were based on the general population rather than the proportion of the population that had these diseases.

SCD was defined as deaths occurring out of the hospital, or in emergency departments, or as “dead on arrival,” with an underlying cause of death reported as a cardiac disease (ICD-10 codes I21-I25) (13,14). Death rates were calculated for Pennsylvania residents aged 35 years or older and standardized to the 2000 US population.

Segmented regression analysis of interrupted time-series method (15) was used to estimate the changes in levels and trends in Pennsylvania adults’ current smoking prevalence, age-adjusted AMI and asthma hospitalization rates, and age-adjusted SCD rates that followed each of the 2 increases in Pennsylvania cigarette excise taxes. This method controls for baseline level and trend when estimating expected changes resulting from increases in Pennsylvania’s cigarette excise tax. The time series regression equation for this analysis is

Ŷ_t_ =β_0_ + β_1 _× Time + β_2i _× Tax_increase_i_ + β_3i _× Time_post_tax_increase_i_ + e_t_

Ŷ_t_ is the independent outcome variable (Pennsylvania adult current smoking prevalence, age-adjusted AMI hospitalization rate, asthma hospitalization rate, and SCD rate). Time is the number of quarters, starting from the first quarter of 1998 as 1, and then increasing by 1 for every quarter thereafter for Pennsylvania adult current smoking prevalence analysis. Age-adjusted AMI and asthma rates analyses, which started with 1 in the first quarter of 2000, increased by 1 for every quarter thereafter. “Tax_increase” is a dummy variable with the value 0 for the segment before the tax increase and 1 for the segment after the tax increase. “Time_post_tax_increase” is the number of quarters after the tax increase, with the value 0 for the segment before the tax increase; subscript *i* equals 1 for July 2002 tax increase and equals 2 for January 2004 tax increase; *e*_t_ is the random variation at time *t* not explained by the model.

The coefficient β_0_ estimates the baseline level of the rates at which Pennsylvania adults currently smoke, are hospitalized for AMI and for asthma, and experience sudden cardiac death at the beginning of the observation; β_1_ estimates the base rate trend before the tax increase; β_2_ estimates the change in rate level after the tax increase. It is the measurement of rate change from the last time point before the tax increase to the first time point after the tax increase; β_3_ estimates the change in rate slope after the tax increase segment.

We calculated the Durbin-Watson statistic to test for the serial autocorrelation of the error terms in the regression models. When autocorrelation existed, we conducted the stepwise autoregression process using the Yule-Walker method with backstep option to correct for autocorrelation. Initial auto-regressive parameters were set to 5 because our data were calculated at the quarterly level to account for seasonality change in rate. All final models had a Durbin-Watson statistic value close to the preferred value of 2. The statistical package SAS version 9.2 (SAS Institute, Cary, North Carolina) was used for all analyses. A *P* value less than .05 was considered significant.

## Results

### Pennsylvania adult current smoking prevalence

No significant changes were seen in current smoking prevalence trend for all adults (aged 18 to 39, or ≥40) during the first quarter of 1998 to the second quarter in 2003 (Table 1a). Likewise, the first tax increase in July 2002 was not associated with a significant change in either the trend or the level for smoking prevalence in all age groups.

**Table 1a:** Interrupted Time-Series Regression Analysis of Adult Smoking Prevalence by Age Category, Pennsylvania, 1998-2010^a^

Variable	All Adults		18–39 y		≥40 y	
	β	*P* Value	β	*P* Value	β	*P* Value
Base level (β_0_)	23.40	<.001	29.95	<.001	19.09	<.001
Base trend (β_1_)	0.07	.30	0.07	.49	0.08	.26
After 1st tax increase change in level (β_21_)	−0.52	.73	−2.35	.33	0.43	.79
After 1st tax increase change in trend (β_31_)	0.21	.54	1.08	.06	−0.22	.55
After 2nd tax increase change in level (β_22_)	−1.75	.14	−5.18	.010	−0.04	.98
After 2nd tax increase change in trend (β_32_)	−0.46	.18	−1.41	.01	0.02	.95

a Adjusted for autocorrelation.

The second tax increase in January 2004 was also not associated with any significant changes in smoking prevalence trend or level for all adults or for adults aged 40 or older. However, significant changes were seen in both the smoking prevalence level and trend for adults aged 18 to 39. The second tax increase was associated with a 5.2% drop in level (*P* = .01) and 1.4% quarterly decrease (*P* = .01) in the smoking prevalence in this younger age group when compared with trend and level before the second tax increase (Table 1a, Figure 1a).

**Figure 1a:**
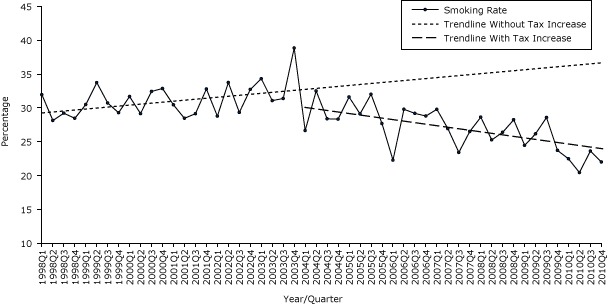
Quarterly smoking prevalence for adults aged 18–39 years, Pennsylvania, 1998–2010. Source: 1998–2010 Behavioral Risk Factor Surveillance System survey data.

**Table:** 

Year/Quarter	Adults Aged 18–39
1998 Q1	31.95
1998 Q2	28.14
1998 Q3	29.22
1998 Q4	28.47
1999 Q1	30.49
1999 Q2	33.73
1999 Q3	30.72
1999 Q4	29.29
2000 Q1	31.68
2000 Q2	29.15
2000 Q3	32.43
2000 Q4	32.86
2001 Q1	30.45
2001 Q2	28.47
2001 Q3	29.14
2001 Q4	32.79
2002 Q1	28.78
2002 Q2	33.75
2002 Q3	29.33
2002 Q4	32.73
2003 Q1	34.31
2003 Q2	31.07
2003 Q3	31.39
2003 Q4	38.86
2004 Q1	26.65
2004 Q2	32.51
2004 Q3	28.39
2004 Q4	28.33
2005 Q1	31.61
2005 Q2	29.11
2005 Q3	32.01
2005 Q4	27.68
2006 Q1	22.27
2006 Q2	29.80
2006 Q3	29.19
2006 Q4	28.80
2007 Q1	29.78
2007 Q2	26.93
2007 Q3	23.41
2007 Q4	26.51
2008 Q1	28.63
2008 Q2	25.27
2008 Q3	26.40
2008 Q4	28.25
2009 Q1	24.46
2009 Q2	26.16
2009 Q3	28.58
2009 Q4	23.72
2010 Q1	22.48
2010 Q2	20.44
2010 Q3	23.61
2010 Q4	22.00

### Age-adjusted acute myocardial infarction hospitalization rate

The age-adjusted AMI hospitalization rate had a significant quarterly decreasing trend of 0.56/100,000 population from the initial value from the first quarter of 2000 (73.80/100,000 population). The first excise tax increase was not associated with changes in either level or trend in the rate of AMI hospitalizations. The second tax increase was associated with a significant reduction in age-adjusted AMI hospitalization rate of 3.22/100,000 population (Table 1b, Figure 1b), though the trend was not significantly changed. When stratified by sex, the age-adjusted 3.87/100,000 population decrease in AMI hospitalizations was strongest and most significant (*P* = .04) for men; the lesser effect for women did not reach significance (Table 1b). Neither tax increase was associated with significant trend changes for AMI hospitalization rates for all adults, men or women.

**Table 1b:** Interrupted Time-Series Regression Analysis of Age-Adjusted AMI Hospitalization Rates by Sex, Pennsylvania, 2000–2009^a^

Variable	All Adults		Male		Female	
	β	*P* Value	β	*P* Value	β	*P* Value
Base level (β_0_)	73.80	<.001	100.29	<.001	52.28	<.001
Base trend (β_1_)	−0.56	.005	−0.84	.006	−0.34	.07
After 1st tax increase change in level (β_21_)	0.01	.99	1.20	.57	−1.22	.55
After1st tax increase change in trend (β_31_)	0.18	.67	0.11	.85	0.39	.39
After 2nd tax increase change in level (β_22_)	−3.22	.02	−3.87	.04	−2.77	.06
After 2nd tax increase change in trend (β_32_)	−0.30	.43	−0.18	.72	−0.55	.21

a Adjusted for autocorrelation.

**Figure 1b:**
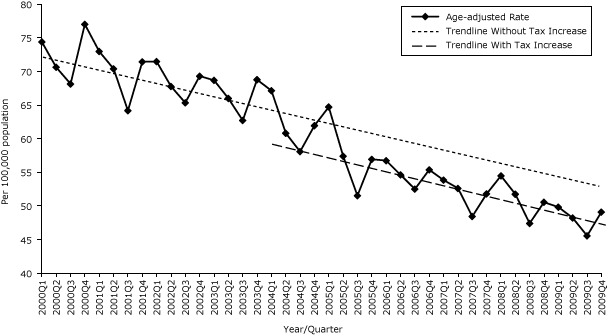
Quarterly age-adjusted AMI hospitalization rates, Pennsylvania, 2000–2009. Source: 2000–2009 Pennsylvania Health Care Cost Containment Council (PHC4) hospital discharge database.

**Table:** 

Year/Quarter	All
2000 Q1	74.39
2000 Q2	70.62
2000 Q3	68.14
2000 Q4	77.00
2001 Q1	72.97
2001 Q2	70.39
2001 Q3	64.18
2001 Q4	71.42
2002 Q1	71.47
2002 Q2	67.77
2002 Q3	65.34
2002 Q4	69.31
2003 Q1	68.69
2003 Q2	66.00
2003 Q3	62.72
2003 Q4	68.78
2004 Q1	67.14
2004 Q2	60.81
2004 Q3	58.09
2004 Q4	61.95
2005 Q1	64.72
2005 Q2	57.40
2005 Q3	51.52
2005 Q4	56.93
2006 Q1	56.73
2006 Q2	54.63
2006 Q3	52.52
2006 Q4	55.38
2007 Q1	53.85
2007 Q2	52.62
2007 Q3	48.45
2007 Q4	51.81
2008 Q1	54.48
2008Q2	51.75
2008 Q3	47.40
2008 Q4	50.56
2009 Q1	49.83
2009 Q2	48.21
2009 Q3	45.56
2009 Q4	49.10

### Age-adjusted and age-specific asthma hospitalization rates

The first excise tax increase was associated with a significant decrease in age-adjusted asthma hospitalizations (6.94/100,000 population [*P* = .01]), but the trend increased (3.02/100,000 population [*P* < .001]). After the second tax increase, significant decreases were seen in both age-adjusted rate (10.05/100,000 population [*P* < .001]) and trend (3.21/100,000 population per quarter) (Table 1c, Figure 1c).

**Table 1c:** Interrupted Time-Series Regression Analysis of Age-Adjusted and Age-Specific Asthma Hospitalization Rates by Age Category, Pennsylvania, 2000–2009^a^

Variable	All Age Groups		≤18 y		18–39 y		≥40 y	
	β	*P* Value	β	*P* Value	β	*P* Value	β	*P* Value
Base level (β_0_)	40.53	<.001	61.42	<.001	26.17	<.001	38.55	<.001
Base trend (β_1_)	0.22	.31	0.04	.97	−0.12	.65	0.37	.48
After 1st tax increase change in level (β_21_)	−6.94	.01	−11.01	.13	−2.37	.34	−4.27	.46
After 1st tax increase change in trend (β_31_)	3.02	<.001	4.88	.01	1.40	.02	3.01	.02
After 2nd tax increase change in level (β_22_)	−10.05	<.001	−22.02	.001	−6.80	.001	−4.39	.28
After 2nd tax increase change in trend (β_32_)	−3.21	<.001	−4.72	.009	−1.29	.02	−3.29	.01

a Adjusted for autocorrelation.

**Figure 1c:**
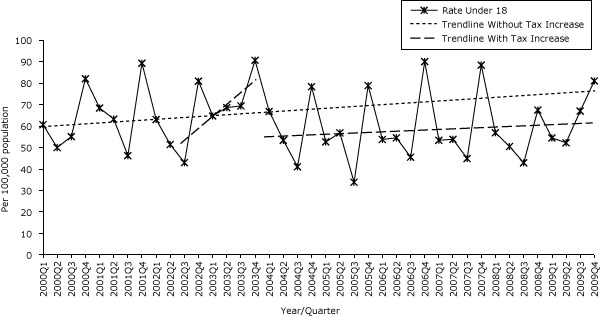
Quarterly age-specific asthma hospitalization rates for children under age 18, Pennsylvania, 2000–2009. Source: 2000–2009 Pennsylvania Health Care Cost Containment Council (PHC4) hospital discharge database.

**Table:** 

Year/Quarter	Under 18 y
2000 Q1	60.62
2000 Q2	49.96
2000 Q3	55.10
2000 Q4	82.03
2001 Q1	68.37
2001 Q2	63.23
2001 Q3	46.24
2001 Q4	89.25
2002 Q1	63.02
2002 Q2	51.42
2002 Q3	42.97
2002 Q4	80.89
2003 Q1	64.70
2003 Q2	68.60
2003 Q3	69.37
2003 Q4	90.61
2004 Q1	66.78
2004 Q2	53.35
2004 Q3	41.04
2004 Q4	78.33
2005 Q1	52.65
2005 Q2	56.83
2005 Q3	33.85
2005 Q4	78.83
2006 Q1	53.74
2006 Q2	54.52
2006 Q3	45.51
2006 Q4	90.00
2007 Q1	53.39
2007 Q2	53.81
2007 Q3	44.87
2007 Q4	88.38
2008 Q1	56.91
2008 Q2	50.50
2008 Q3	42.89
2008 Q4	67.46
2009 Q1	54.45
2009 Q2	52.21
2009 Q3	66.99
2009 Q4	81.01

No significant changes in the age-specific asthma hospitalization rate levels were seen for any age group — under 18, aged 18 to 39, or aged 40 or older — but the trends all increased after the first excise tax increase. The second excise tax increase was associated with both a decreased rate level and decreasing trend for age-specific asthma hospitalization rate in the age groups under 18 and 18 to 39, with the most notable decrease for children aged 18 years or younger (22.02/100,000 population [*P* < .001]) and trend (4.72/100,000 population per quarter [*P* < .001]). The asthma hospitalization rate for adults aged 40 or older demonstrated a decreasing trend after the second tax increase but had no significant decrease in level.

### Age-adjusted sudden cardiac death rate

Since 2000, the overall age-adjusted SCD rate decreased significantly for all adults, both men and women. The overall age-adjusted SCD rate for all adults was associated with an increasing trend after the first tax increase but no significant change in rate level. The second tax increase was associated with a reduction in the level of 0.72/100,000 population in overall SCD. The SCD rate for men was associated with a significant decrease of 1.34/100,000 population in level with the second tax increase but was not associated with any significant change in trend. No significant changes were seen in women (Table 1d, Figure 1d).

**Table 1d:** Interrupted Time-Series Regression Analysis of Age-Adjusted Sudden Cardiac Death Rates by Sex, Pennsylvania, 2000–2009^a^

Variable	All Adults		Male		Female	
	β	*P* Value	β	*P* Value	β	*P* Value
Base level (β_0_)	19.03	<.001	28.16	<.001	12.58	<.001
Base trend (β_1_)	−0.23	<.001	−0.33	<.001	−0.17	<.001
After 1st tax increase change in level (β_21_)	−0.01	.85	0.07	.56	−0.08	.43
After 1st tax increase change in trend (β_31_)	0.81	.01	1.05	.06	0.57	.20
After 2nd tax increase change in level (β_22_)	−0.72	.001	−1.34	<.001	−0.13	.67
After 2nd tax increase change in trend (β_32_)	0.12	.08	0.09	.44	0.15	.10

a Adjusted for autocorrelation.

**Figure 1d:**
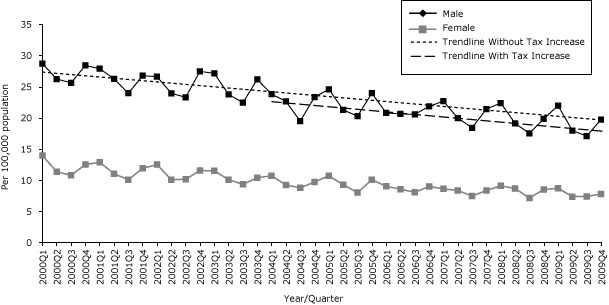
Quarterly age-adjusted sudden cardiac death (SCD) rates by sex, Pennsylvania, 2000–2009. Source: Pennsylvania 2000–2009 Vital Statistics death certificate data.

**Table:** 

Year/Quarter	Male	Female
2000 Q1	28.63	13.94
2000 Q2	26.16	11.33
2000 Q3	25.55	10.78
2000 Q4	28.33	12.49
2001 Q1	27.81	12.86
2001 Q2	26.19	11.02
2001 Q3	23.89	10.08
2001 Q4	26.68	11.89
2002 Q1	26.54	12.49
2002 Q2	23.88	10.08
2002 Q3	23.23	10.15
2002 Q4	27.38	11.53
2003 Q1	27.06	11.51
2003 Q2	23.69	10.07
2003 Q3	22.38	9.34
2003 Q4	26.11	10.38
2004 Q1	23.73	10.70
2004 Q2	22.59	9.24
2004 Q3	19.43	8.77
2004 Q4	23.26	9.71
2005 Q1	24.52	10.69
2005 Q2	21.23	9.27
2005 Q3	20.22	8.01
2005 Q4	23.89	10.07
2006 Q1	20.74	9.03
2006 Q2	20.58	8.56
2006 Q3	20.51	8.05
2006 Q4	21.80	9.00
2007 Q1	22.62	8.61
2007 Q2	19.93	8.35
2007 Q3	18.37	7.47
2007 Q4	21.36	8.35
2008 Q1	22.32	9.09
2008 Q2	19.07	8.67
2008 Q3	17.48	7.16
2008 Q4	19.78	8.51
2009 Q1	21.92	8.71
2009 Q2	17.93	7.37
2009 Q3	17.05	7.40
2009 Q4	19.67	7.79

## Discussion

The Pennsylvania cigarette excise tax increases were found to be effective in decreasing smoking prevalence only among adults aged 18 to 39 after the second tax, which increased the price to more than $4.00/pack. After each tax increase, cigarette consumption decreased, according to the number of packs of cigarettes sold (11). In other words, despite the increase in per-pack tax, the actual state tax revenues from cigarettes sales decreased (11). To our knowledge, no studies have evaluated the effectiveness of the Pennsylvania excise tax increases on reducing adult smoking prevalence or on adverse health effects. 

Our findings are consistent with those of the National Academy of Sciences’ IOM 1998 report “Taking Action to Reduce Tobacco Use” (16) and its updated 2007 report “Ending the Tobacco Problem” (8), which conclude that the single most direct and reliable method for reducing tobacco consumption is to increase the price of tobacco products. The National Cancer Institute Expert Panel’s report provides relevant information regarding the influence of both smoking prevalence and number of cigarettes smoked on cigarette consumption (6). That is, a 10% increase in cigarette price has been found to reduce overall cigarette consumption by approximately 3% to 5%; approximately two-thirds of this reduction in consumption is attributed to individuals choosing not to smoke at all (6). Our findings support a recent study that reported the inverse, namely, that reductions in cigarette consumption are mainly due to reductions in consumption by smokers (64%) rather than reductions in smoking prevalence (36%) (17).

A review of Pennsylvania’s cigarette price history reported the average per pack price in 2001 was $3.20 with $0.65 per pack overall tax (18). The average per pack price increased $0.78 to $3.98 in 2002 after the first Pennsylvania cigarette excise tax increase of $0.69. However, the price per pack further rose $0.32 in 2004 to $4.30 when the second excise tax increased $0.35 to $1.74 overall tax (18). One should also be cognizant that tobacco companies may adjust prices to absorb part of the tax increase so it is not passed to the consumer (19).

The significant decline in smoking prevalence among adults aged 18 to 39 after the second excise tax increase may be the result of the overall per pack price ($4.30) finally exceeding the psychological tipping point of $4.00 per pack. Our findings provide additional evidence to support the IOM reports (8,16) that it is not the cigarette tax itself but the overall price resulting from the tax increase that may prompt changes in smoking prevalence and trends. Younger smokers, especially those under age 20, are much more sensitive to overall price than older smokers. Older adults are likely to have smoked longer and are more likely to demonstrate long-standing addictive behaviors that are more difficult to modify. Older adults are also more likely to be able to financially adjust to cigarette price increases and thus have a higher tipping point for smoking cessation. This is consistent with results from other studies that indicate cigarette excise tax increases have a greater impact on younger adults than on older adults because of their economic status (7). Cigarette taxes and effects of price increases on the smoking habits of older adults need to be studied further.

Our findings are consistent with previous studies that show a positive association between smoking and trends in AMI and asthma (20–27). Although the second tax increase did not significantly alter the trend in the AMI hospitalization rate, it did appear to reset the trend line, producing a 3.22/100,000 population overall rate reduction. This translates into more than 350 fewer AMI hospitalizations per year in Pennsylvania. In 2009, the average AMI hospitalization length of stay was 5.25 days and the average hospitalization charge was $71,057 in Pennsylvania (PHC4 data). This correlates with approximately 2,000 fewer hospitalization days and an estimated savings of $25 million in hospital charges. There was also a significant reduction in the level of the age-adjusted SCD rate following the second tax increase for men but not for women. This may simply reflect the greater burden of SCD in men than women. This apparent paradox, of no significant change in smoking prevalence among the older at-risk adults corresponding to a reduction in AMI hospitalization rates and SCD, may be partially explained by the decrease in cigarette consumption by all adults (11) (rather than decrease in smoking prevalence) and reduced secondhand smoke exposure.

The first tax increase was associated with a significant decrease in the level but an increase in the trend of asthma-associated hospitalizations. The second tax increase was associated with further decreases in the level and reversed the trend of the asthma hospitalization rate. These changes were much more pronounced in younger age groups, especially in those under 18 years of age. These age-specific differences in the impact of the increased excise tax may be related to the decline in smoking prevalence among young adults aged 18 to 39 who no longer expose their children under 18 years old to secondhand smoke.

A major strength of our analytical approach is that we used the more rigorous interrupted time-series method (15), which controls for baseline level and trend when estimating the changes due to Pennsylvania cigarette excise tax increases. This quasi-experimental design is preferred over the simpler pre- and postproportion comparison method (23,27) that does not take the preintervention trend into consideration. The conclusions may be flawed in pre–post analyses because there is no accounting for the preintervention overall trend that would have continued without the intervention. We also found evidence of autocorrelation in the data and adjusted with Yule-Walker autoregression method, which usually is not considered in simpler pre-post proportional analysis. Another strength of our analytic approach is that we used hospital discharge data that include all cases rather than a sample. In addition, our smoking prevalence estimates represent the smoking prevalence in Pennsylvania because they were derived from BRFSS, a well-designed multiple stage stratified survey designed to represent the state. The use of multiple robust data sources adds to one’s confidence in the validity of our findings.

There are several limitations of this analysis. We examined the relationship of the excise tax increases on only 3 conditions. These conditions were chosen because they are known to be affected by smoking in a short term. However, AMIs and SCDs are rare in adults aged 18 to 39, and asthma is less common in older adults. This may have limited the likelihood that the associations with excise tax increases would reach significance. Second, this is an ecological explorative study and causal inferences cannot be strictly made. The study was based on overall state rates with no specific control group, but the interrupted time-series method compares both trend and level pre-tax and post-tax, controlling for all other unknown factors (15). Other factors temporally related to the excise tax increases may influence smoking prevalence and could potentially inflate the effectiveness of the cigarette tax and price effect. We examined this possibility but did not find any significant health program changes during the study period. Third, medical treatment and other improvements in the prevention and management of AMI and asthma could not be addressed in the analysis. However, it is unlikely that these improvements would be so closely aligned with the excise tax changes that were the basis for the analysis. Health insurance coverage and access to health care and hospitals could also influence our findings but they could not be assessed in this analysis.

Although the impact of the excise taxes on overall smoking prevalence were not as dramatic as predicted from other studies, the analysis in this study suggests that the increase in excise tax, along with the increase in price, decreased smoking prevalence in younger adults and was positively correlated with changes in asthma hospitalizations, especially in the young, and with the occurrence of SCD and AMI hospitalizations in men. Additional research and policy development is needed to assess the effectiveness of further cigarette taxes in decreasing 1) tobacco consumption (smoking prevalence and amount of cigarettes smoked) while being cognizant of psychological tipping points for price; and 2) smoking-related health outcomes and health care costs.
